# Abnormal coagulation after hepatectomy in patients with normal preoperative coagulation function

**DOI:** 10.1186/s12893-024-02406-2

**Published:** 2024-05-06

**Authors:** Liting Kuang, Weibin Lin, Dahui Wang, Bin Chen

**Affiliations:** 1 Department of Anesthesiology, the First Afflicted Hospital of Sun Yet-sen University, Guangzhou, 510080 China; 2 Department of Cardiac Surgery, the First Affiliated Hospital of Sun Yat-Sen University, No.58 Zhongshan II Road, Guangzhou, 510080 Guangdong China; 3 Department of Liver Surgery, the First Affiliated Hospital of Sun Yat-sen University, Guangzhou, China

**Keywords:** Postoperative abnormal coagulation, Risk factors, INR, Succinyl gelatin

## Abstract

**Background:**

To explore the risk factors for postoperative abnormal coagulation (PAC) and establish a predictive model for patients with normal preoperative coagulation function who underwent hepatectomy.

**Materials and Methods:**

A total of 661 patients with normal preoperative coagulation function who underwent hepatectomy between January 2015 and December 2021 at the First Affiliated Hospital of Sun Yat-sen University were divided into two groups: the postoperative abnormal coagulation group (PAC group, *n* = 362) and the normal coagulation group (non-PAC group, *n* = 299). Univariate and multivariate logistic analyses were used to identify the risk factors for PAC.

**Results:**

The incidence of PAC in 661 patients who underwent hepatectomy was 54.8% (362/661). The least absolute shrinkage and selection operator (LASSO) method was used for multivariate logistic regression analysis. The preoperative international normalized ratio (INR), intraoperative succinyl gelatin infusion and major hepatectomy were found to be independent risk factors for PAC. A nomogram for predicting the PAC after hepatectomy was constructed. The model presented a receiver operating characteristic (ROC) curve of 0.742 (95% confidence interval (CI): 0.697–0.786) in the training cohort. The validation set demonstrated a promising ROC of 0.711 (95% CI: 0.639–0.783), and the calibration curve closely approximated the true incidence. Decision curve analysis (DCA) was performed to assess the clinical usefulness of the predictive model. The risk of PAC increased when the preoperative international normalized ratio (INR) was greater than 1.025 and the volume of intraoperative succinyl gelatin infusion was greater than 1500 ml.

**Conclusion:**

The PAC is closely related to the preoperative INR, intraoperative succinyl gelatin infusion and major hepatectomy. A three-factor prediction model was successfully established for predicting the PAC after hepatectomy.

## Introduction

Hepatocellular carcinoma is still one of the most common tumors in the world, and hepatectomy is the main treatment for hepatocellular carcinoma [1]. Bleeding and thromboembolism are major complications following hepatectomy; hence, early anticoagulation to avoid venous thromboembolism (VTE) is critical [2]. However, effective analgesia for abdominal surgery can hasten recovery. However, many investigators do not use epidural analgesia or early anticoagulation due to concerns about postoperative abnormal coagulation (PAC). If the risk of PAC can be detected and controlled, the use of epidural analgesia is safer, and anticoagulation can be initiated early postoperatively. Postoperative analgesia and anticoagulation will improve, as will perioperative complications. Many studies have identified preoperative cirrhosis, preoperative coagulation disorders, preoperative platelet levels, hepatectomy, and intraoperative bleeding as risk factors for PAC following hepatectomy [3–7]. Some studies have shown that the incidence of PAC after hepatectomy is relatively high, possibly due to a variety of factors, such as the patient’s preoperative liver dysfunction, the degree of intraoperative blood loss, the residual liver volume, and ischemia‒reperfusion injury [6, 8, 9]. Early PAC might increase postoperative bleeding, postoperative complications, or hospital stays in patients with other diseases [10, 11]. However, the PAC appears to be self-limiting in patients who undergo hepatectomy [2]. Moreover, no risk factor studies have been conducted in individuals with normal preoperative coagulation who underwent hepatectomy in China. The purpose of this retrospective study was to explore the risk factors for impaired coagulation function following hepatectomy for hepatocellular cancer in China.

## Materials and methods

### Patients and ethics

**Fig. 1:**
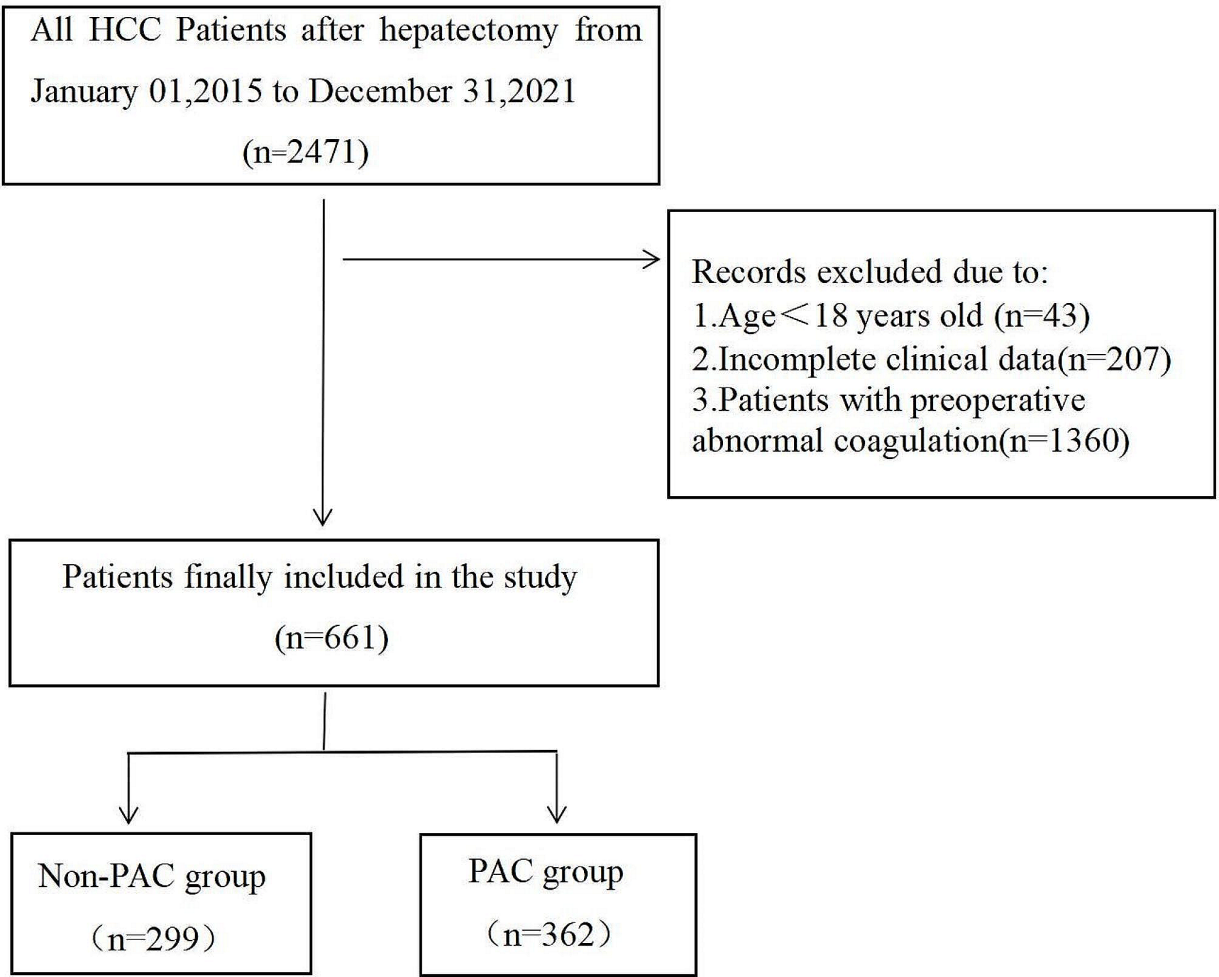
Flow chart of the study

From January 2015 to December 2021, 661 patients who underwent hepatectomy for hepatocellular carcinoma at the First Affiliated Hospital of Sun Yat-sen University had normal preoperative coagulation function, and all enrolled patients had complete preoperative examination data and a coagulation function review within 48 h of surgery. The inclusion criteria were as follows: (1) aged ≥ 18 years, American Society of Anesthesiologists (ASA) I-III; and (2) had a normal preoperative peripheral blood coagulation function index. The exclusion criteria were patients who (1) had preoperative coagulation disorders, (2) had incomplete clinical data, or (3) consumed coagulation-altering medicines within one week prior to surgery. (4) Major cardiovascular illness, (5) aberrant renal function, (6) other cancers or metastasis to other organs, and (7) immune system disease. (8) Patients with a recent history of abnormal bleeding. (9) Allergy to plasma, gelatin, or starch. Our study complied with the relevant provisions of ethics and adhered to the Declaration of Helsinki, as described in the text below. The study was approved by the ethics committee of the First Affiliated Hospital of Sun Yat-sen University on May 30, 2023 (No. [2023]198). The data were completely anonymized to remove any identifying information, and the need for informed consent was waived. The study complied with all regulations. The flow chart of the study is shown in Fig. 1.

### Definition of PAC

The definition of preoperative normal coagulation function must simultaneously meet the following conditions: 11 s ≤ prothrombin time (PT) ≤ 14 s, 80%≤PT%≤130%, 0.80 ≤ INR ≤ 1.15, 25 s ≤ activated partial thromboplastin time (APTT) ≤ 31.2 s, 14 s ≤ thrombin time (TT) ≤ 21 s, 1.8 g ≤ fibrinogen (FIB) ≤ 3.5 g, and platelet count ≥ 100 × 10^9^/L. Postoperative coagulation abnormalities were defined as the presence of any of the following disorders within 48 h after surgery [7]: an INR ≥ 1.4, a PLT < 80 × 10^9^/L, or an APTT > 38 s. The recruited patients were divided into two groups based on the above criteria: those with abnormal coagulation (PAC group, *n* = 362) and those with normal coagulation (non-PAC group, *n* = 299).

### Data collection

We retrospectively reviewed the medical history, surgical records, anesthesia records, and examination tests of all patients who underwent hepatectomy for hepatocellular carcinoma at our institution from January 2015 to December 2021. Age, sex, body mass index (BMI), past medical history, preoperative imaging, laboratory tests, preoperative medications, intraoperative medications, intraoperative blood and fluid transfusions, surgical modality, duration of surgery, and intraoperative low central venous pressure time were all collected retrospectively from medical history, surgical records, anesthesia records, and examination tests.

### Surgical procedures

Before surgery, the potential liver and tumor status were evaluated through enhanced computed tomography (CT), magnetic resonance imaging (MRI), or abdominal ultrasound. Patients with severe liver fibrosis or other organ dysfunction will have a residual liver volume of at least 40% in the future, and patients with normal liver function are allowed to have a residual liver volume of > 30%. In the process of hepatectomy, the Pringle maneuver was used to block blood flow into the liver when necessary. Major hepatectomy was defined as resection of at least three hepatic segments [3]. Intermittent clamping was performed as patterns of alternating clamping phase and reperfusion phase: 15-5-15-5.Under anesthesia, low central venous pressure method was applied to reduce liver volume perfusion and minimize blood loss.

### Statistical analysis

All the statistical analyses were performed with Stata/SE 17.0 (College Station, TX77845, USA) and R version 4.2.2 software (https://www.r-project.org/). The normality of the distribution of the data was assessed using the Kolmogorov–Smirnov test. Continuous variables are reported as the mean ± standard deviation (SD) and were compared using Student’s independent t test. Nonnormally distributed data are reported as the median [interquartile range (IQR)] and were tested by a nonparametric test (Mann‒Whitney *U* test). Categorical variables are presented as numbers and percentages [n(%)] and were compared using the χ2 test or Fisher’s exact test (if an expected value ≤ 5 was found).

All datasets were randomly divided into training and validation cohorts at a 7:3 ratio, and homogeneity analysis was performed between the two cohorts. The least absolute shrinkage and selection operator (LASSO) method was used to reduce the number of candidate predictors. The penalty term was determined by 10-fold cross-validation, selecting the penalty that yielded the smallest mean square error. The optimal model, with the fewest variables, was identified based on λ = 1se as the criterion. These variables were subjected to multivariate analysis to calculate odds ratios (ORs) and *P* values. Factors with *P* < 0.05 in the multivariate analysis were included in the subsequent analysis. The variables that were significant in the multivariate logistic regression model were recognized as variables associated with PAC and were used in the final model.

A nomogram was also established by the final model through the “rms” package of R software. The predicted probabilities of the models were evaluated by the receiver operating characteristic (ROC) curve and area under the curve (AUC). Calibration plots (1000 bootstrap resamples) and the Hosmer‒Lemeshow goodness-of-fit test (HL test) were used to evaluate the accuracy of the prediction models, and *P* > 0.05 indicated that the model calibration degree was reliable. Decision curve analysis (DCA) was performed to assess the clinical usefulness of the rmda package in R. A two-tailed *P* < 0.05 was considered to indicate statistical significance. The research flowchart is shown in Fig. 1.

## Results

### Incidence of PAC

From January 2015 to December 2021, the incidence of PAC was 54.8% (362/661). PAC was detected in 160 patients with an INR ≥ 1.4, 124 patients with an APTT > 38 s, and 83 patients with a PLT < 80 × 10^9^/L.

### Comparison of preoperative data and intraoperative conditions

Compared with those in the validation cohort, patients in the training cohort had no significant differences in preoperative indicators or intraoperative conditions (*P* > 0.05). There was good homogeneity between the training cohort and validation cohort in terms of patient demographic and clinical characteristics (Table 1 and 2).

**Table 1:** Comparison of preoperative baseline characteristics in training cohort and the validation cohort

Parameters	All (*n* = 661)	Training cohort(*n* = 465)	Validation cohort(*n* = 196)	*P*
**General characteristics**				
PAC (%)				0.766
Yes	362 (54.8)	253 (54.4)	109 (55.6)	
No	299 (45.2)	212 (45.6)	87 (44.4)	
Age, y	55.06 ± 11.38	55.13 ± 11.48	54.88 ± 11.16	0.800
Gender, (%)				0.599
Male	554 (83.8)	392 (84.3)	162 (82.7)	
Female	107 (16.2)	73 (15.7)	34 (17.3)	
BMI,, Kg/m^2^	23.16 ± 3.19	22.84 ± 3.20	22.96 ± 3.14	0.256
ASA grade, (%)				0.717
1	29 (4.4)	19 (4.1)	10 (5.1)	
2	505 (76.4)	359 (77.2)	146 (74.5)	
3	127 (19.2)	87 (18.7)	40 (20.4)	
MELD score	17.34 ± 1.82	17.32 ± 1.76	17.38 ± 1.95	0.686
ALBI score	-2.57 ± 0.31	-2.57 ± 0.31	-2.59 ± 0.30	0.430
APRI score	0.61 ± 0.61	0.60 ± 0.59	0.64 ± 0.66	0.465
FIB-4 score	2.22 ± 1.90	2.23 ± 2.09	2.19 ± 1.37	0.804
Tumor diameter, cm	6.06 ± 4.02	6.05 ± 4.29	6.08 ± 3.29	0.922
**Preoperative complications**				
HBV infection, n (%)				0.099
Yes	515(77.9)	368(79.1)	147(75.0)	
No	146(22.1)	97(20.9)	49(25.0)	
HP, n (%)				0.899
Yes	103 (15.6)	73 (15.7)	30 (15.3)	
No	558 (84.4)	392 (84.3)	166 (84.7)	
CAD, No. (%)				0.384
Yes	18 (2.7)	11 (2.4)	7 (3.6)	
No	643 (97.3)	454 (97.6)	189 (96.4)	
Diabetes, n (%)				0.532
Yes	54 ( 8.2)	40 (8.6)	14 (7.1)	
No	607 (91.8)	425 (91.4)	182 (92.9)	
HL, n (%)				0.655
Yes	59 (8.9)	43 (9.2)	16 (8.2)	
No	602 (91.1)	422 (90.8)	180 (91.8)	
COPD, No. (%)				0.484
Yes	18 (2.7)	14 (3.0)	4 (2.0)	
No	643 (97.3)	451 (97.0)	192 (98.0)	
Liver cirrhosis, n (%)				0.656
Yes	181 (27.4)	125 (26.9)	56 (28.6)	
No	480 (72.6)	340 (73.1)	140 (71.4)	
**Laboratory measurements**				
Hb, g/L	141.25 ± 17.75	141.04 ± 17.74	141.76 ± 17.80	0.634
PLT,×10^9^/L	189.67 ± 58.97	189.98 ± 59.92	188.72 ± 56.80	0.803
ALB, g/L	39.34 ± 3.67	39.23 ± 3.62	39.60 ± 3.77	0.245
TBIL,µmol/L	16.92 ± 15.00	16.64 ± 14.97	17.56 ± 15.07	0.245
UREA,µmol/L	4.99 ± 1.66	5.04 ± 1.79	4.87 ± 1.31	0.225
CREA,µmol/L	78.55 ± 33.29	79.58 ± 38.52	76.13 ± 14.59	0.224
ALT, U/L	30.0 (20.0, 44.0)	30.0(19.0,46.3)	30.0(20.0,44.0)	0.573
AST, U/L	32.0 (24.0, 45.0)	34.0(25.0,48.0)	32.0(24.0,44.0)	0.349
GGT, U/L	54.0 (32.0, 105.0)	56.0(34.0,108.5)	54.0(31.0,104.0)	0.528
LDH, U/L	200.0 (172.0, 233.0)	199.0(172.0,236.3)	200.0(172.0,231.0)	0.848
ALP, U/L	82.0 (69.0, 104.0)	83.5(70.0,105.8)	80.0(68.0,103.0)	0.176
CHE, U/L	6711 ± 1552	6738 ± 1646	6648 ± 1306	0.496
PT, s	11.89 ± 0.55	11.89 ± 0.54	11.87 ± 0.58	0.633
PT%	93.46 ± 8.09	93.30 ± 7.86	93.82 ± 8.63	0.452
INR	1.02 ± 0.05	1.02 ± 0.05	1.01 ± 0.05	0.152
APTT, s	28.01 ± 1.86	27.95 ± 1.74	28.15 ± 2.12	0.218
TT, s	18.44 ± 1.17	18.46 ± 1.19	18.42 ± 1.14	0.706
FIB, g/L	2.65 ± 1.17	2.67 ± 0.47	2.60 ± 0.46	0.093
**Preoperative medication**				
ACEI/ARB, n (%)				0.600
Yes	13 (2.0)	10 (2.2)	3 (1.5)	
No	648 (98.0)	455 (97.8)	193 (98.5)	
CCB, n (%)				0.627
Yes	77 (11.6)	56 (12.0)	21 (10.7)	
No	584 (88.4)	409 (88.0)	175 (89.3)	
Preoperative Nsaids, n(%)				0.272
Yes	17 (2.6)	14 (3.0)	3 (1.5)	
No	644 (97.4)	451 (97.0)	193 (98.5)	
Statin (%)				0.328
Yes	9 (1.4)	5 (1.1)	4 (2.0)	
No	652 (98.6)	460 (98.9)	192 (98.0)	
Diuretics, n (%)		192 (98.0)	454 (97.6)	0.798
Yes	15 (2.3)	4 (2.0)	11 (2.4)	
No	646 (97.7)	1354 (95.6)	581 (96.2)	
Hormones, n (%)				0.109
Yes	7 (1.1)	3 (0.6)	4 (2.0)	
No	654 (98.9)	462 (99.4)	192 (98.0)	

Abbreviations: ACEI/ARB: Angiotensin-converting enzyme inhibitor/Angiotensin II receptor blocker; ALB: Albumin; ALBI: albumin-bilirubin; ALP: Alkaline phosphatase; ALT: Alanine transaminase; APRI, AST to Platelet Ratio Index; APTT: Activated partial thromboplastin time; AST: Aspartate aminotransferase; BMI, Body mass index; CAD, coronary artery disease; CCB: Calcium channel blocker; CHE: Cholinesterase; COPD: chronic obstructive pulmonary diseases; CREA: Creatinine; FIB: Fibrinogen; FIB-4, fibrosis4; GGT: γ-glutamyl transpeptidase; HBV: Hepatitis B Virus; Hb: Hemoglobin; HL, Hyperlipidemia; HP, Hypertension; INR: International normalized ratio; LDH: Lactic Dehydrogenase; MELD: Model for end-stage liver disease; NSAIDs, non-steroidal anti-inflammatory drugs; PAC: Postoperative abnormal coagulation; PLT: Platelet; PT: Prothrombin time; PT%: Prothrombin activity; TBIL: Total bilirubin; TT: Thrombin time; UREA: Blood urea nitrogen

**Table 2:** Comparison of intraoperative conditions in training cohort and the validation cohort

Parameters	All (*n* = 661)	Training cohort(*n* = 465)	Validation cohort(*n* = 196)	*P*
**Surgical and anesthetic Factors**				
Surgery time, minute	240.27 ± 95.05	241.85 ± 96.96	236.52 ± 90.48	0.510
Sevoflurane inhalation, No. (%)				0.875
Yes	578 (87.4)	406 (87.3)	172 (87.8)	
No	83 (12.6)	59 (12.7)	24 (12.2)	
Dexmedetomidine, No. (%)				0.578
Yes	517 (78.2)	361 (77.6)	156 (79.6)	
No	144 (21.8)	104 (22.4)	40 (20.4)	
Intraoperative NSAIDs, No. (%)				0.581
Yes	377 (57.0)	262 (56.3)	115 (58.7)	
No	284 (43.0)	203 (43.7)	81 (41.3)	
Dopamine, No. (%)				0.568
Yes	120 (18.2)	87 (18.7)	33 (16.8)	
No	541 (81.8)	378 (81.3)	163 (83.2)	
Norepinephrine, No. (%)				0.805
Yes	278 (42.1)	197 (42.4)	81 (41.3)	
No	383 (57.9)	268 (57.6)	115 (58.7)	
Major hepatectomy, n (%)				0.428
Yes	193 (29.2)	140 (30.1)	53 (27.0)	
No	468 (70.8)	325 (69.9)	143 (73.0)	
Low CVP time, min	143.77 ± 138.84	144.88 ± 104.07	141.12 ± 198.66	0.751
Minimum SBP, mmHg	81.10 ± 9.89	81.22 ± 10.30	80.81 ± 8.87	0.620
Minimum MAP, mmHg	57.49 ± 7.34	57.37 ± 7.35	57.76 ± 7.33	0.534
Epidural anesthesia, n (%)				0.822
Yes	53 (8.0)	38 (8.2)	15 (7.7)	
No	608 (92.0)	427 (91.8)	181 (92.3)	
Estimated blood loss, ml	250 0.0(100.0, 600.0)	200 0.0(100.0, 500.0)	300 0.0(100.0, 600.0)	0.312
**Intra-operative insfusion**				
Crystalloids infusion, ml	2214.92 ± 847.98	2243.12 ± 850.84	2295.92 ± 808.60	0.460
Hydroxyethyl starch infusion, ml	0.0(0.0, 500.0)	0 0.0(0.0, 500.0)	0 0.0(0.0, 500.0)	0.288
Succinyl gelatin infusion, ml	500.0(0.0, 1000.0)	500.0(0.0, 1000.0)	500.0(0.0, 1000.0)	0.922
Albumin, ml	28.74 ± 58.83	29.89 ± 60.96	26.02 ± 53.46	0.440
RBC, ml	138.59 ± 433.75	140.54 ± 414.47	133.95 ± 477.48	0.859
Plasma, ml	80.26 ± 241.57	83.98 ± 230.97	71.43 ± 265.45	0.542
Total infusion, ml	3381.86 ± 1661.24	3377.10 ± 1681.17	3393.14 ± 1617.15	0.910
Insfusion per hour per Kg, ml	10.27 ± 3.99	10.15 ± 4.06	10.56 ± 3.81	0.236

Abbreviations: MAP, mean artery pressure; RBC, red blood cell; SBP, systolic blood pressure

### Risk factors for PAC and multivariable logistic regression analysis

**Fig. 2:**
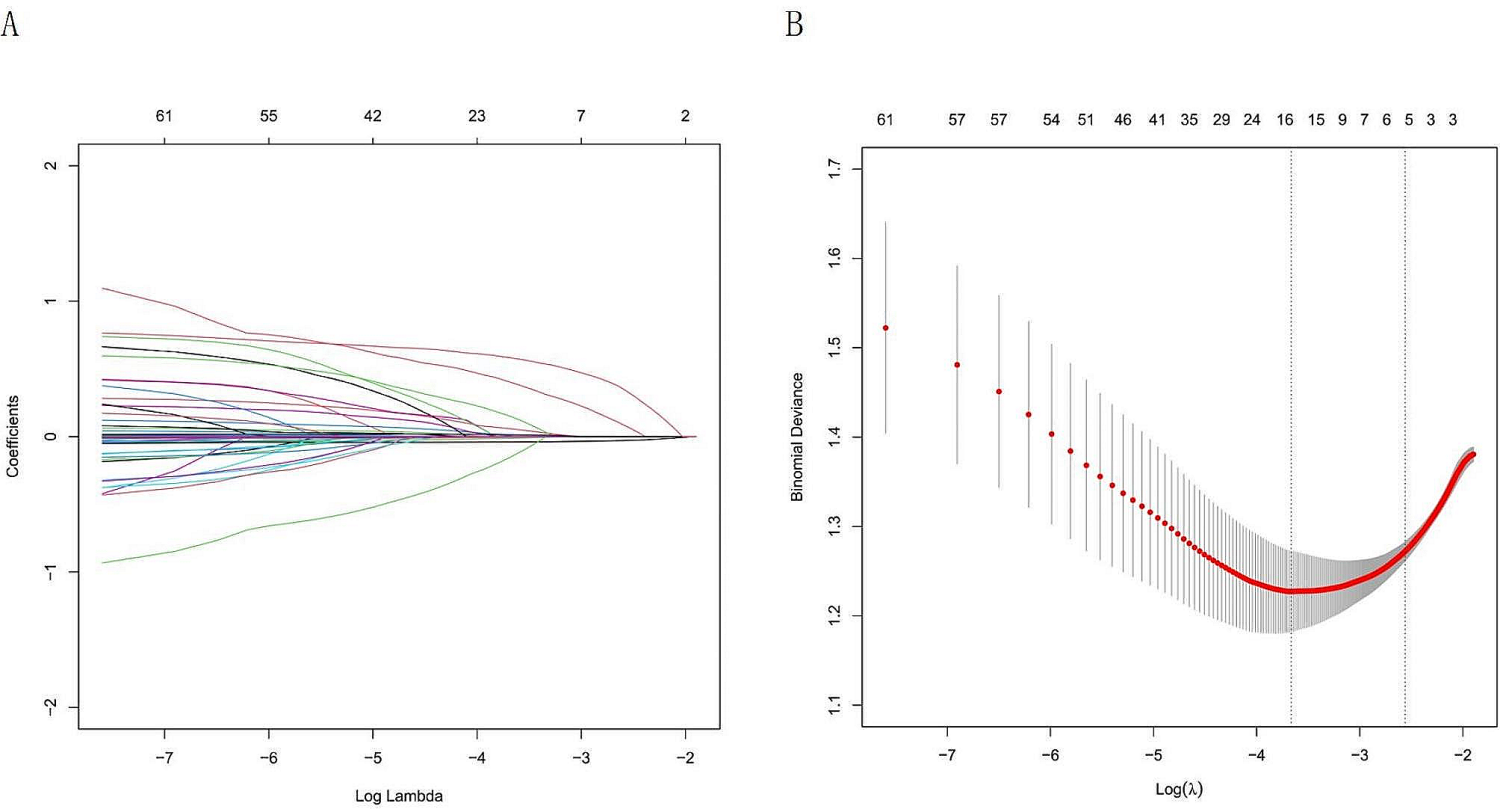
Selection of PAC patient features using the LASSO logistic regression model. (**A**) A Lasso coefficient profile plot was built for the prediction of PAC. (**B**) The optimal parameter (λ) was selected by the LASSO model using 10-fold cross-validation via 1 standard error of the minimum criteria

To further remove the confounding factors in Tables 1 and 2, the variables of preoperative indicators and intraoperative conditions were subjected to LASSO regression analysis. Five risk factors, namely, the preoperative international normalized ratio (INR), estimated blood loss, major hepatectomy, succinyl gelatin infusion and red blood cell (RBC) infusion, were identified by using lambda.1 se = 0.0754 (Fig. 2A and B). By analyzing these five variables via multivariable logistic regression analysis, we found that preoperative INR (OR: 3.648; 95% CI: 2.378 to 5.710), major hepatectomy (OR: 2.117; 95% CI: 1.152 to 3.901), and succinyl gelatin infusion (OR: 1.0005; 95% CI: 1.0001 to 1.0009) were found to be independent risk factors for PAC (*P* < 0.05) (Table 3).

**Table 3:** The univariate and multivariate logistic regression model of independent variables to PAC

Parameters	Univariate logistic			Multivariate logistic	
	OR (95% CI)	*P*		OR (95% CI)	*P*
INR×10	3.249 (2.183,4.914)	< 0.001		3.648 (2.378,5.710)	< 0.001
Estimated blood loss	1.0011 (1.0007,1.0016)	< 0.001		1.0005 (1.0001,1.0009)	0.246
Major hepatectomy	3.588 (2.329,5.632)	< 0.001		2.117 (1.152,3.901)	0.016
Succinyl gelatin infusion	1.0008(1.0007,1.0016)	< 0.001		1.0005 (1.0001,1.0009)	0.025
RBC infusion	1.002(1.001,1.003)	< 0.001		1.001 (1.000,1.002)	0.147

### Construction of the nomogram column chart model

**Fig. 3:**
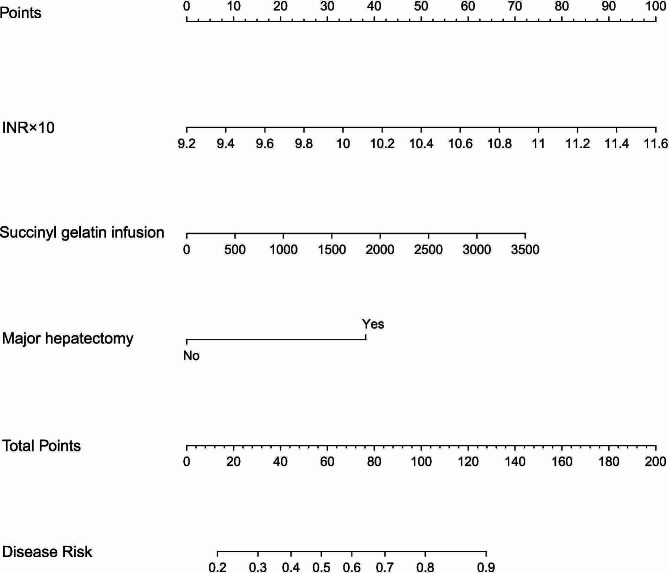
The nomogram of factors associated with the final model for diagnosing intraoperative MBT, including preoperative INR, intraoperative succinyl gelatin infusion, and major hepatectomy. (To use the nomogram, an individual patient’s value is located on each variable axis, and a line is drawn upward to find the points received for each variable value. Then, the sum of these numbers is located on the total points axis, and a line is drawn downward to the risk of the PAC axes to determine the likelihood of PAC).

We used the three independent risk factors to construct a nomogram column chart model. Each patient can be scored based on the identified risk factors. The higher the total score is, the greater the possibility of PAC. This score enabled us to develop a preliminary prediction for the possibility of PAC (Fig. 3).

### Internal validation of the ROC curve

**Fig. 4:**
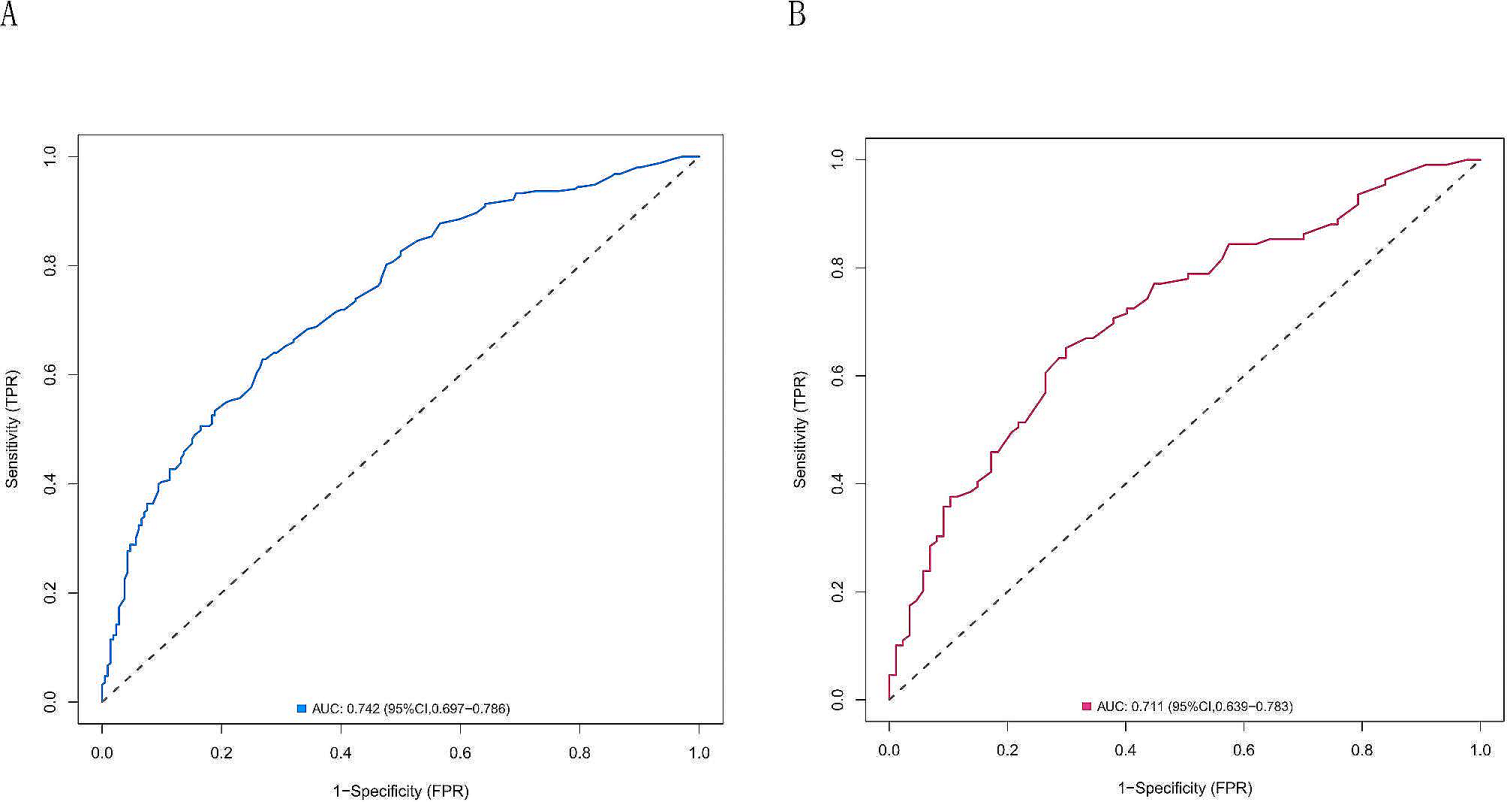
Receiver operating characteristic (ROC) curves of the model for predicting PAC. (**A**) ROC curve of the prediction model in the training cohort. (**B**) ROC curve of the prediction model in the validation cohort

ROC curves were constructed for the training cohort and validation cohort to evaluate the predictive models. The area under the curve (AUC) of the training cohort was 0.742 (95% CI: 0.697–0.786, *P* < 0.001), and the sensitivity, specificity, positive predictive value (PPV), negative predictive value (NPV) and Youden’s index were 0.628, 0.731, 0.736, 0.622 and 0.360, respectively (Fig. 4A). For the validation cohort, the AUC was 0.711 (95% CI: 0.639–0.783, *P* < 0.001), and the sensitivity, specificity, PPV, NPV and Youden’s index were 0.596, 0.736, 0.739, 0.593 and 0.332, respectively (Fig. 4B). There was no significant difference between the validation and training cohorts (*P* > 0.05).

### Internal validation of the Hosmer‒Lemeshow (H-L) goodness-of-fit calibration test

**Fig. 5:**
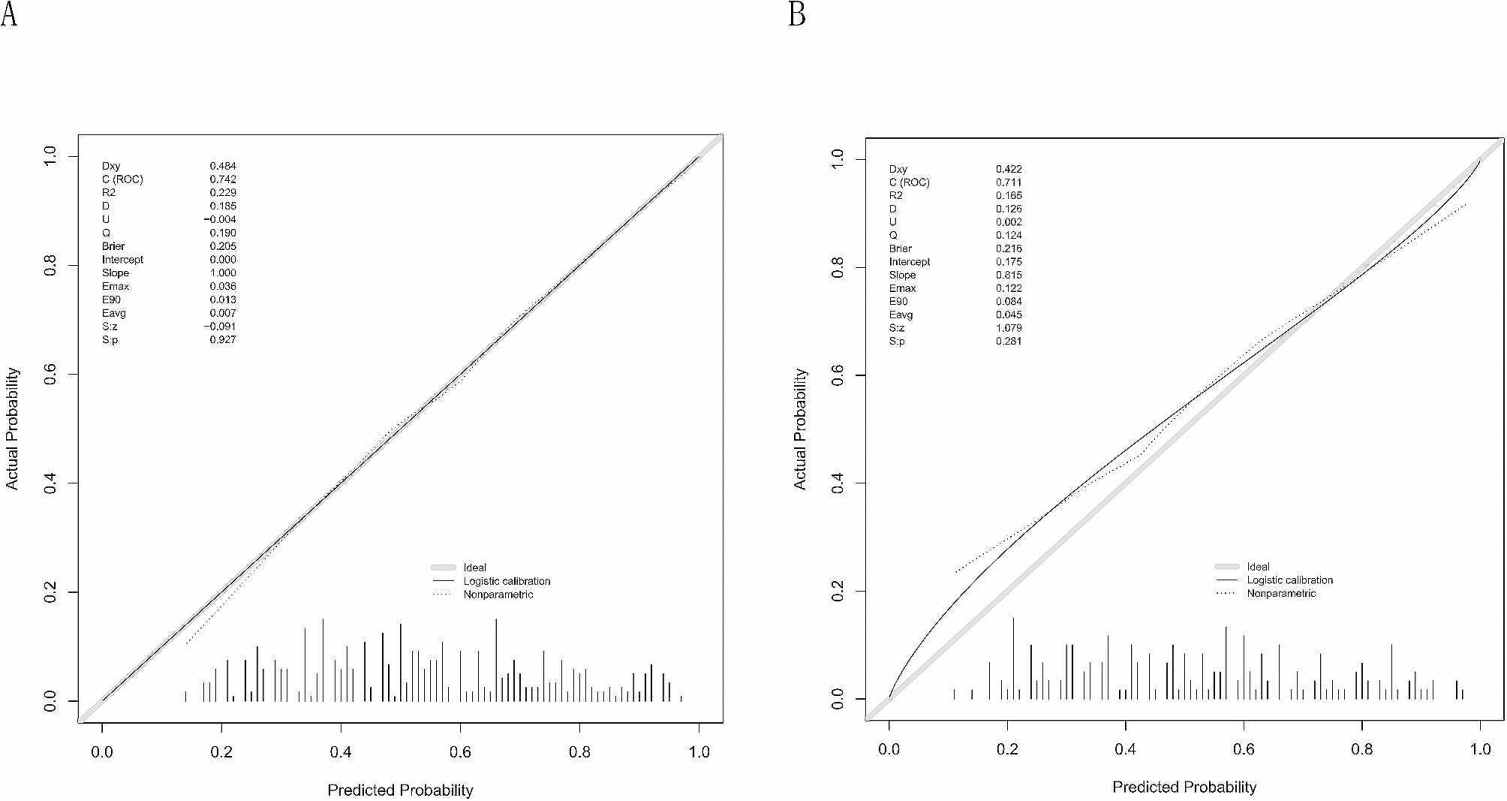
Calibration plots for the model in predicting PAC. (**A**) Calibration plot of the nomogram in the training cohort. (**B**) Calibration plot of the nomogram in the validation cohort

For the training cohort, the C-index was 0.741 (χ2 = 12.792, *P* = 0.804) (Fig. 5A); for the validation cohort, the C-index was 0.710 (χ2 = 11.251, *P* = 0.883) (Fig. 5B). The calibration curve slopes for both the training cohort and the validation cohort were close to 1, indicating strong agreement between the model and the actual risk and a high predictive value.

### Decision curve analyses to assess clinical utility

**Fig. 6:**
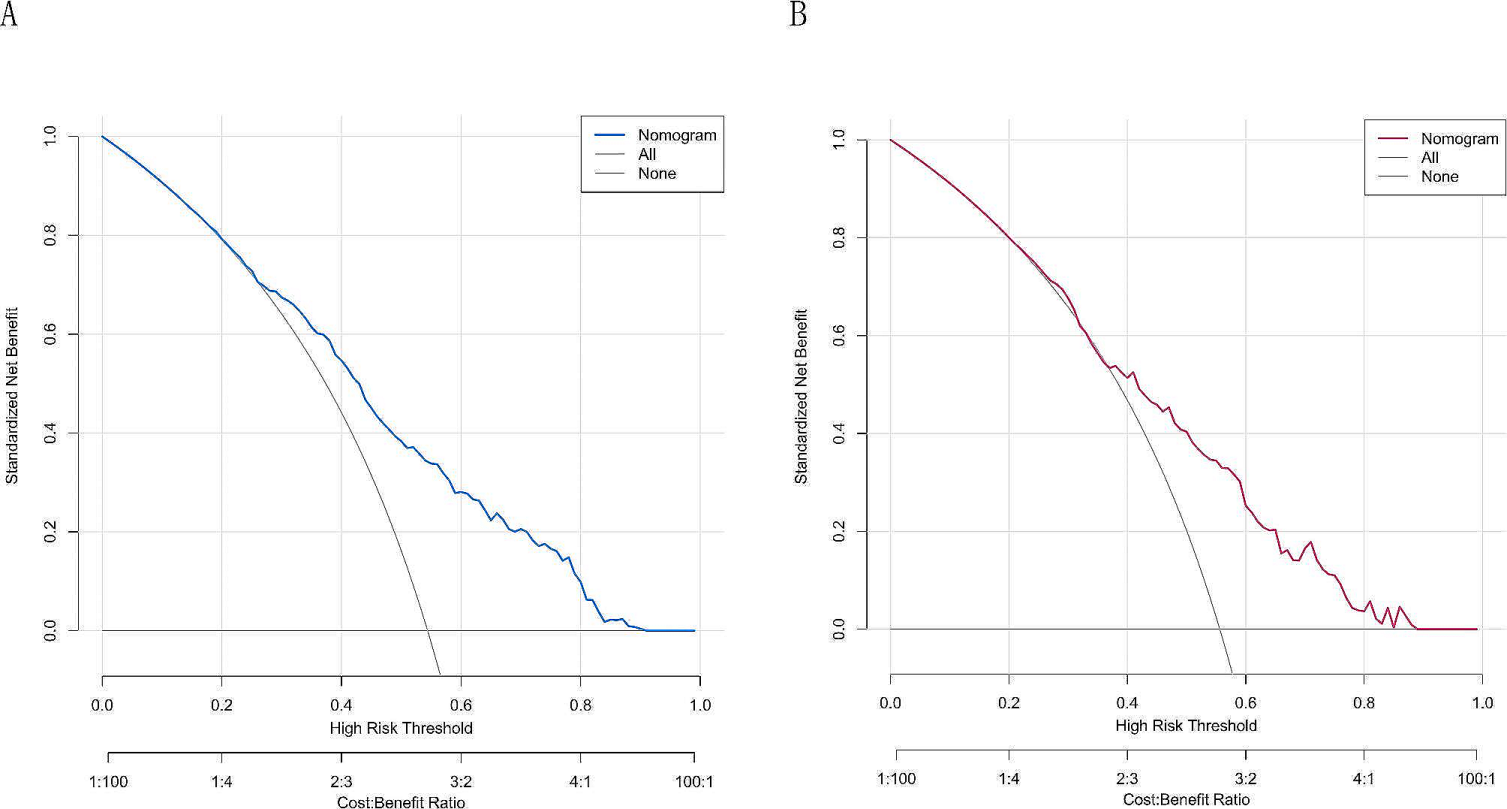
Decision curve analysis (DCA) comparing the net benefit of the model in predicting PAC. (**A**) DCA plot of the nomogram in the training cohort. (**B**) DCA plot of the nomogram in the validation cohort

The DCA of the nomogram model is presented in Fig. 6A and B. The DCA curves provided a favorable net benefit across a wide range, with a threshold probability across 30–90% and 40–90% for the training and validation cohorts, respectively.

### Restricted cubic splines (RCSs) between preoperative INR, succinyl gelatin infusion and PAC

**Fig. 7:**
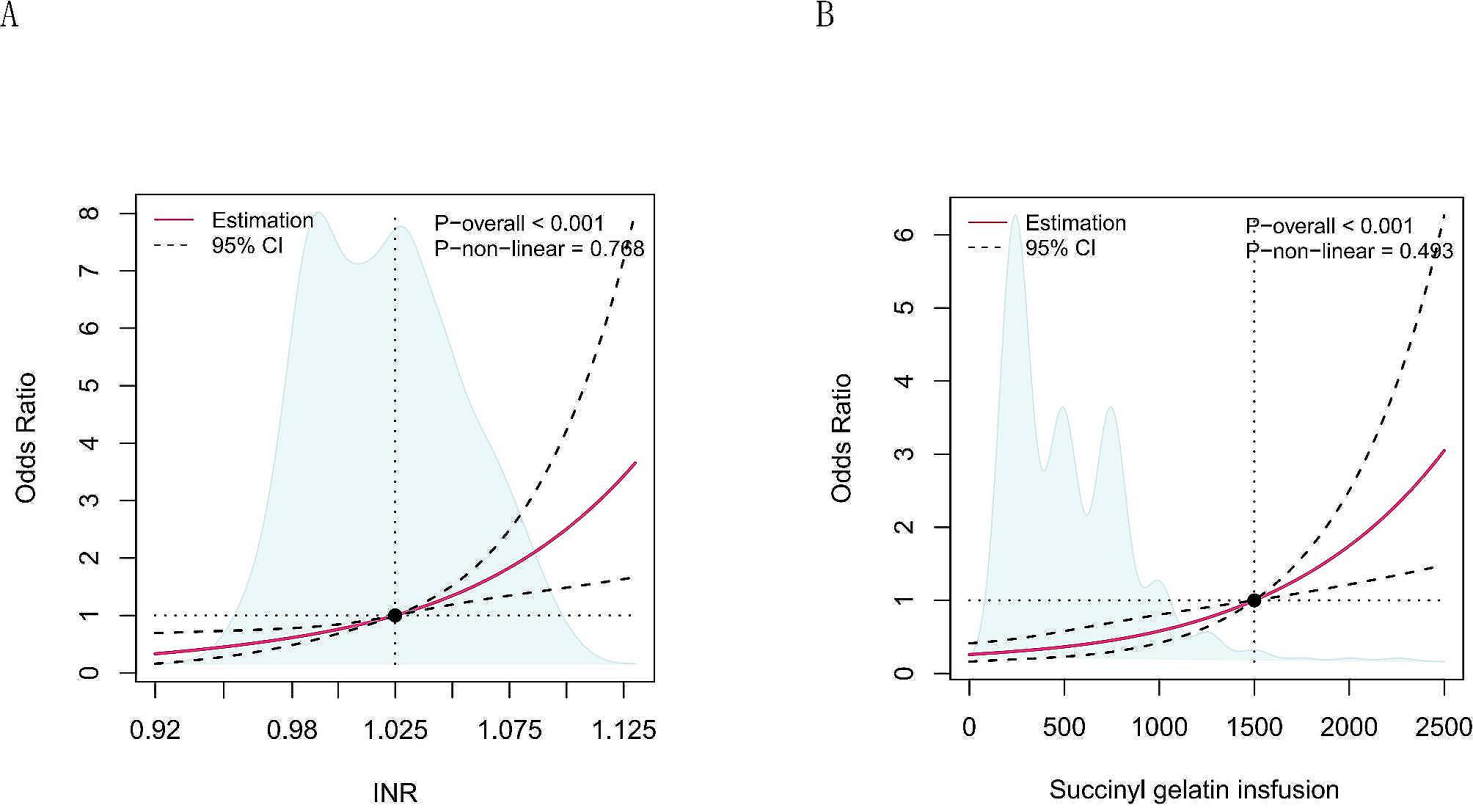
Restricted cubic splines between preoperative INR (**A**), intraoperative succinyl gelatin infusion (**B**) and PAC. Solid red lines are multivariable adjusted odds ratios, with dashed bold lines showing 95% confidence intervals derived from restricted cubic spline regressions. The reference lines for no association are indicated by the black dashed lines at a hazard ratio of 1.0

The RCS confirmed a linear association between the preoperative INR (*P*-overall < 0.001, *P-*nonlinear = 0.768; Fig. 7A), the incidence of succinyl gelatin infusion (*P*-overall < 0.001, *P-*nonlinear = 0.493; Fig. 7B) and the PAC. The reference points for the preoperative INR and amount of succinyl gelatin infusion were 1.025 and 1500 ml, respectively. There was a linear relationship between preoperative INR, succinyl gelatin infusion and PAC. The risk of PAC increased linearly when the preoperative INR was above 1.025 and the amount of succinyl gelatin infusion was above 1500 ml.

## Discussion

Even in individuals with normal preoperative coagulation, the frequency of postoperative abnormal coagulation was not low, according to our findings. Preoperative INR, intraoperative succinyl gelatin infusion and major hepatectomy were found to be independent risk factors for coagulopathy after hepatectomy. Patients with preexisting coagulation abnormalities combined with intraoperative bleeding and hepatectomy were indeed more likely to have postoperative coagulation abnormalities. However, in patients with normal preoperative coagulation, we found risk factors for abnormal postoperative coagulation.

Abnormal coagulation is prevalent following hepatectomy for hepatocellular carcinoma, and malfunction of liver parenchymal cells can lead to coagulation dysfunction, even in individuals who have fairly normal coagulation function before surgery. In our study, the incidence of abnormal coagulation after hepatectomy was 54.8%, which was greater than that in healthy living donors [12].

Although some studies did not include the INR, preoperative coagulation problems remain an important risk factor for posthepatectomy abnormal coagulation [6]. In Tina et al.’s study, the preoperative INRs without postoperative coagulation abnormalities and transient and persistent coagulation abnormalities were 1.05 ± 0.06, 1.10 ± 0.09, and 1.15 ± 0.08, respectively [7]. As shown, even small changes in the INR prior to surgery were significant risk factors. As a result, preparing products such as platelets, cold precipitates, and prothrombin complexes prior to hepatectomy may help with coagulation problems.

No difference between 40 and 70% hepatectomy was found when coagulopathy was evaluated in an animal study [13]. However, major hepatectomy has been a risk factor in different clinical studies. Decreased coagulation factor levels are strongly related to major hepatectomy [6, 14]. Major hepatectomies frequently result in excessive bleeding and necessitate intraoperative infusions of red blood cells and plasma. Red blood cell transfusions might result in inappropriate postoperative coagulation [15, 16]. This is consistent with our findings.

There is currently controversy over whether succinyl gelatin causes abnormal coagulation. Our study indicated that while succinyl gelatin also served as an intraoperative plasma substitute, it appeared to be more prone to coagulation disorders than the other components. When blood products are not yet available in the case of substantial intraoperative blood loss, the transfusion of albumin and artificial colloids (hydroxyethyl starch, succinyl gelatin, etc.) is frequently necessary. Although succinyl gelatin was formerly assumed to have no effect on clotting other than through dilution, there is now mounting evidence that succinyl gelatin does have an effect on platelet function and clotting. Thromboelastography and scanning electron microscopy were used to investigate in vitro coagulation in the presence of succinyl gelatin, where the weight and strength of the clots generated in the presence of succinyl gelatin were decreased and the usual reticular network of fibrin chains was lost. After succinyl gelatin administration, thrombin generation was reduced, presumably due to hemodilution [17, 18]. Kam et al. reported that hemodilution of more than 20% with succinyl gelatin impaired coagulation more strongly than that observed with NS hemodilution [19]. Palmaers et al. reported that gelatin significantly inhibited whole blood coagulation and platelet function in neurosurgical procedures [20]. Only succinyl gelatin was identified as a risk factor for abnormal coagulation following hepatectomy in our study, which may be related to the fact that succinyl gelatin infusion is frequently chosen over hydroxyethyl starch in situations of excessive bleeding.

Since the incidence of bleeding was lower in both groups than in other studies, heavy bleeding is a risk factor for abnormal postoperative coagulation. Thus, improved surgical techniques and reduced bleeding can help improve postoperative abnormal coagulation.

The findings of this study provide a clinical reference for predicting postoperative coagulation problems in hepatectomy patients with hepatocellular carcinoma, although there are significant limitations. First, this was a retrospective study in which data were obtained during hospitalization, and only changes in coagulation function within 48 h of surgery were noted, with no long-term follow-up or follow-up. Second, while arterial blood pressure is continuously monitored during most hepatectomies, it is only recorded at 5-minute intervals on electronic anesthesia sheets, so we cannot assess the duration of hypotension in each patient or pinpoint the precise stage at which blood pressure is at its lowest. Third, this study concentrated on preoperative and intraoperative risk factors for coagulation disorders, with little emphasis on postoperative risk variables. Fourth, we did not determine how many patients with PAC needed to be treated. Fifth, the postoperative disease processes of PAC patients with different INRs were not compared.

## Conclusion

Postoperative coagulation problems following hepatectomy are associated with a variety of preoperative and intraoperative risk factors, and more complete screening and monitoring are required to minimize the occurrence of postoperative abnormal coagulation and improve patient prognosis.

## Data Availability

The datasets used and analysed during the current study are available from the corresponding author on reasonable request.

## Abbreviations

PAC: Postoperative abnormal coagulation
LASSO: Least absolute shrinkage and selection operator
INR: International Normalized Ratio
ROC: Receiver operating characteristic
CI: Confidence interval
DCA: Decision curve analysis
VTE: Venous Thromboembolism
ASA: American Society of Anesthesiologists
PT: Prothrombin time
APTT: Activated partial thromboplastin time
TT: Thrombin time
FIB: Fibrinogen
BMI: Body mass index
CT: Computed tomography
MRI: Magnetic resonance imaging
OR: Odds ratio
RCS: Restricted cubic splines
PPV: Positive predictive value
NPV: Negative predictive value
